# A Simulation Study of Carrier Capture Ability of the Last InGaN Quantum Well with Different Indium Content for Yellow-Light-Emitting InGaN/GaN Multiple Quantum Wells

**DOI:** 10.3390/mi14091669

**Published:** 2023-08-26

**Authors:** Wei Liu, Zeyu Liu, Hengyan Zhao, Junjie Gao

**Affiliations:** School of Microelectronics, Northwestern Polytechnical University, Xi’an 710072, China

**Keywords:** last InGaN quantum well, yellow light, In content, carrier capture, droop effect

## Abstract

Currently, GaN-based blue- and green-light-emitting devices have achieved successful applications in practice, while the luminescence efficiency of devices with longer wavelengths (such as yellow light) is still very low. Therefore, in this paper, the electroluminescence characterization of yellow-light-emitting InGaN/GaN multiple quantum wells (MQWs) with different In content in the last InGaN quantum well, which is next to the p-type GaN electrode layer, are investigated numerically to reveal a possible physical mechanism by which the different distribution of In content in the active region impacts the carrier capture and the light emission process in yellow InGaN/GaN MQWs. The simulation results show that at low injection currents, the luminescence efficiency of high-In-content yellow MQWs is enhanced, which can be ascribed to the enhanced radiative recombination process induced by the increased carrier concentration in the last InGaN quantum wells with promoted carrier capture ability. However, in the case of high injection condition, the luminescence efficiency of yellow MQWs deteriorates with increasing In content, i.e., the droop effect becomes remarkable. This can be ascribed to both significantly enhanced Auger recombination and electron leakage in the last InGaN quantum well, induced also by the promoted capture ability of charge carriers.

## 1. Introduction

Group III-nitride semiconductor materials have been widely used in various fields such as lighting and display due to their wide range of emission wavelengths, fast response, high energy efficiency and environmental protection [1]. At present, blue- and green-light-emitting devices, e.g., light-emitting diodes (LEDs) and laser diodes (LDs) based on InGaN/GaN multiple quantum wells (MQWs) have been successfully commercialized, while the luminescence efficiency of GaN-based LEDs with long wavelengths (such as yellow light) is still very low. There are a number of factors contributing to the low efficiency of yellow LEDs. First, in high-In-content InGaN QWs, the quantum confined Stark effect (QCSE) caused by polarization electric fields is enhanced, reducing the luminescence efficiency [2]. Second, during the growth of the high-In-content InGaN well layer in MQWs, misfit dislocations and stacking faults, as well as other crystal defects, are prone to occur due to the aggravated lattice mismatch. These defects may act as non-radiative recombination centers, further reducing the efficiency of yellow LED devices [3]. In addition, under high injection currents, as the potential well of high-In-content InGaN QW is deeper, it is easy to cause an accumulation of a large number of carriers, which may lead to serious Auger recombination [4] and carrier leakage [5]. This makes the luminescence efficiency significantly reduced at high currents, resulting in an enhancement of the so-called droop effect for yellow LEDs [6]. Therefore, it is currently a hot topic to study and optimize the long-wavelength luminescence efficiency of GaN-based LEDs in this field. At present, many measures are proposed to effectively improve the luminescence efficiency of long-wavelength LEDs, such as inserting a stress buffer layer [7,8,9], changing the thickness of the active layer [10], using non-polar surface growth devices [11], growing gradually varying indium (In) content quantum wells [12], using C-doped GaN film as a current spreading layer [13], and so on. On the other hand, researchers are also deepening their research on the relevant physical mechanisms involved in the long-wavelength (yellow) GaN-based LEDs. For example, some have reported that Auger recombination is the main reason for the enhanced droop effect of yellow LEDs under high current [14], while some others explored the reasons which are responsible for the improved luminescence efficiency of yellow GaN-based LEDs by using InGaN barriers and modified electron injection layers [15]. 

In fact, for yellow-light-emitting InGaN/GaN multiple quantum wells (MQWs), the depth of the quantum potential well of InGaN layers is relatively deeper due to the relatively higher In content in InGaN layers compared to the conventional blue InGaN QWs. Therefore, during the electroluminescence (EL) process, combined with the notable difference in mobility between electrons and holes, most electrically injected electrons and holes can be easily accumulated in the last InGaN quantum well (LIQW), which is the closest InGaN QW to the P-type region [16]. As a result, the luminescence emission is mainly contributed by the carrier radiative recombination in the LIQW. Therefore, in this paper, the effect of In content in the LIQW on the performance of yellow InGaN/GaN MQWs is carefully investigated by analyzing the internal quantum efficiency, distribution of carrier concentration, radiative recombination rates, and so on. It is found that for the high-In-content sample, the carrier capture ability of the LIQW can be enhanced due to the deepened depth of the potential well with increased In content, thus improving the luminescence efficiency of yellow MQWs at low injection currents. However, in the case of high injection conditions, the luminescence efficiency deteriorates remarkably with increasing In content in the LIQW, i.e., the droop effect is enhanced, which may be attributed to the significantly enhanced Auger recombination and electron leakage.

## 2. Sample Structure and Simulation Parameters

**Figure 1:**
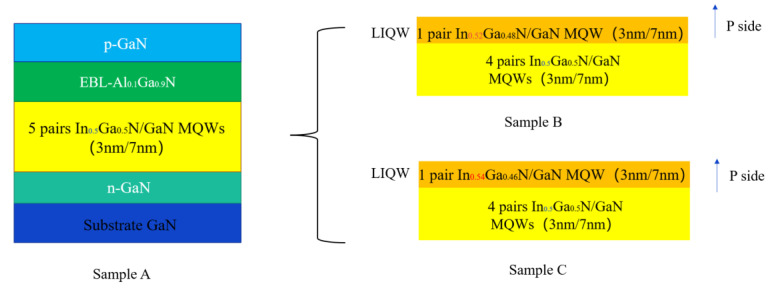
Schematic diagram of yellow InGaN/GaN MQW samples.

The epitaxial structure of the yellow InGaN/GaN MQW sample is mainly composed of the following four parts: the bottom layer is a 200 nm thick N-type GaN substrate with a Si-doping concentration of $1\times 10^{18}{\;\mathrm{cm}}^{-3}$, the InGaN/GaN MQW active region, a 20 nm thick AlGaN electron blocking layer (EBL) with Al content of 10%, and finally a 200 nm thick P-type GaN cap layer with Mg-doping concentration of $1\times 10^{18}{\;\mathrm{cm}}^{-3}$. The MQW active region consists of five pairs of 3 nm thick InGaN quantum well layers and 7 nm thick GaN quantum barrier layers, which are periodically alternated and repeated. The EL characteristics of yellow MQW samples were studied by using a vertical structure for the injection of electrons and holes. The positive and negative electrodes are located above the P-type GaN cap layer and below the N-type GaN substrate, respectively. When a forward bias is applied, electrons and holes can be directly injected into the MQW active region from the N-type and P-type GaN layers, respectively. The epitaxial structures were identical for all samples, except that the In content in the LIQW was varied. The In content in the LIQW was set to be 50%, 52%, and 54% for samples A, B, and C, respectively, while they were all kept at 50% for the rest of the InGaN QWs in the MQW region. In other words, in our study, only the potential well depth of the LIQW was gradually deepened from samples A to C. All samples’ structures are schematically shown in Figure 1.

In this work, the simulation models of the designed MQW structures were calculated by using a three-band wurtzite k·p model, which was performed using SILVACO TCAD software [17]. The carrier recombination models included the general radiative recombination model (OPTR), Shockley–Read–Hall recombination model (SRH), and Auger recombination model (AUGER). 

The OPTR model mainly describes the radiative recombination and generation of photons, and the radiative recombination rate is calculated using the following formula [18]:

(1) $$R_{np}^{OPT}=C_{c}^{OPT}\left(np-n_{ie}^{2}\right)$$

where $R_{np}^{OPT}$ is the radiative recombination rate; $n_{ie}$ is the intrinsic carrier concentration; and $C_{c}^{OPT}$ is the capture rate of the material, which was set as $2\times 10^{-11}{\;\mathrm{cm}}^{3}/s$ [19]. 

The SRH model describes the Shockley–Read–Hall non-radiative recombination in quantum wells, and the SRH recombination rate is calculated using the following formula [18]:

(2) $$R^{SRH}=\frac{pn-n_{ie}^{2}}{\tau_{p0}\left[n+n_{ie}exp\left(\frac{E_{TRAP}}{kT_{L}}\right)\right]+\tau_{n0}\left[p+n_{ie}exp\left(\frac{-E_{TRAP}}{kT_{L}}\right)\right]}$$

In the equation, $E_{TRAP}$ is the difference between the intrinsic Fermi levels and the trap energy level; $T_{L}$ is the lattice temperature in Kelvin degrees; p, n represent the excess hole or electron concentration, respectively; $n_{ie}$ is the intrinsic carrier concentration; and $\tau_{p0}$ and $\tau_{n0}$ represent the lifetimes of holes and electrons, respectively. 

The AUGER model describes Auger recombination, and the Auger recombination rate is calculated using the following formula [18]:

(3) $$R_{Auger}=C_{AUGN}\left(pn^{2}-nn_{ie}^{2}\right)+C_{AUGP}\left(np^{2}-pn_{ie}^{2}\right)$$

In the equation, $C_{AUGN}$ and $C_{AUGP}$ are the Auger coefficients of electrons and holes, which were set to be $1.5\times 10^{-30}{\;\mathrm{cm}}^{6}/s$ in this study [19]. The numerical solution method uses the coupling solution process of Poisson and Schrodinger equations, as well as the current continuity equation and carrier drift–diffusion transfer equation [20].

Finally, it should be noted that for the actual epitaxial growth process, lattice defects and interfacial irregularities may occur in the InGaN/GaN MQWs, resulting in a deteriorated crystal quality. This may cause the non-radiative SRH recombination process, which can be characterized by the SRH carrier recombination lifetime. Therefore, during our simulation, for simplification, it was assumed that the crystal quality was the same for all the simulated MQW samples, and correspondingly, the non-radiative SRH recombination processes were identical for all samples studied here. Therefore, the SRH lifetime was set to be the same value of 100 ns for all InGaN/GaN MQW samples in our work [19]. 

## 3. Results and Discussion

**Figure 2:**
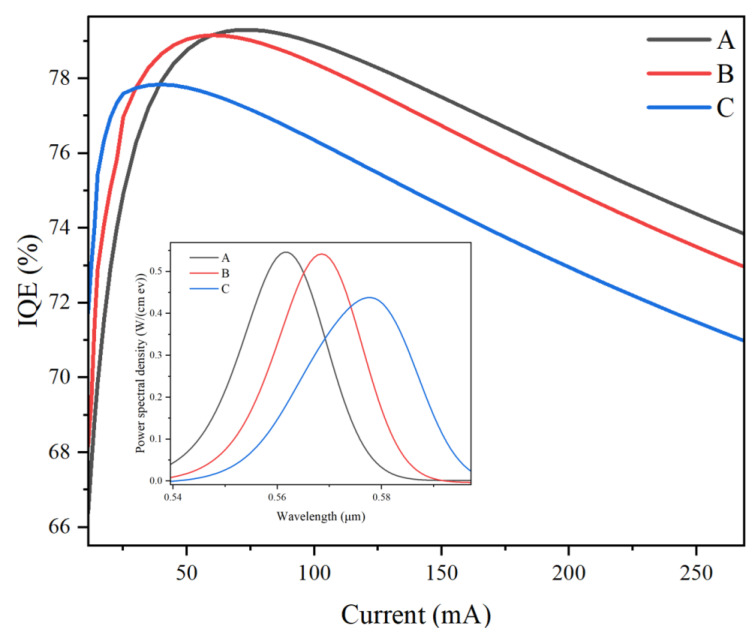
Variation of IQE values with increasing injection currents for all yellow MQW samples. The inset shows the EL spectra of all samples at 150 mA injection current.

Figure 2 shows the value of internal quantum efficiency (IQE) as a function of injection currents for all yellow MQW samples. It can be observed that, in general, the IQE values decreased with increases in the In content in the LIQW from samples A to C. However, on the contrary, it was found that at low injection currents, e.g., below about 20 mA, the IQE values increased from samples A to sample C. On the other hand, when the injection current increased above 100 mA, all samples’ IQE values decreased remarkably, i.e., the droop effect occurred for all MQW samples. In particular, the droop effect became more significant for sample C with the highest In content in the LIQW. In short, when the In content increases in the LIQW, the sample’s EL efficiency is improved at low currents, but it is reduced more seriously at high currents, which will be discussed in detail later. 

The inset in Figure 2 shows the EL spectra at an injection current of 150 mA for all samples. It can be seen that the spectral peaks of all samples are located in the yellow light region at about 560–580 nm and it is obvious that the EL peak wavelength redshifts from samples A to C, which can be ascribed to the reduction of energy bandgap of InGaN alloy and the enhancement of polarization-induced QCSE due to the increased In content in the LIQW [21]. It was also found that the EL spectral width of sample C was much larger than the two others. This may be attributed to the fact that in sample C, the In content of the LIQW was 54%, while it was 50% for the other InGaN QWs. It is well known that the In content in InGaN QWs determines the EL peak wavelength. Therefore, for sample C, with significantly different In content between the LIQW and the other four QWs, the EL spectrum of entire MQWs can be broadened, resulting in an increased spectral width and even a weak bimodal emission. In addition, at 150 mA injection current, the integrated EL intensities of samples A, B, and C were 11.55, 11.45, and 11.26, respectively. From samples A to C, it can be seen that the reduction of integrated EL intensity at 150 mA injection current is consistent with the decrease in IQE values at high currents in Figure 2. 

**Figure 3:**
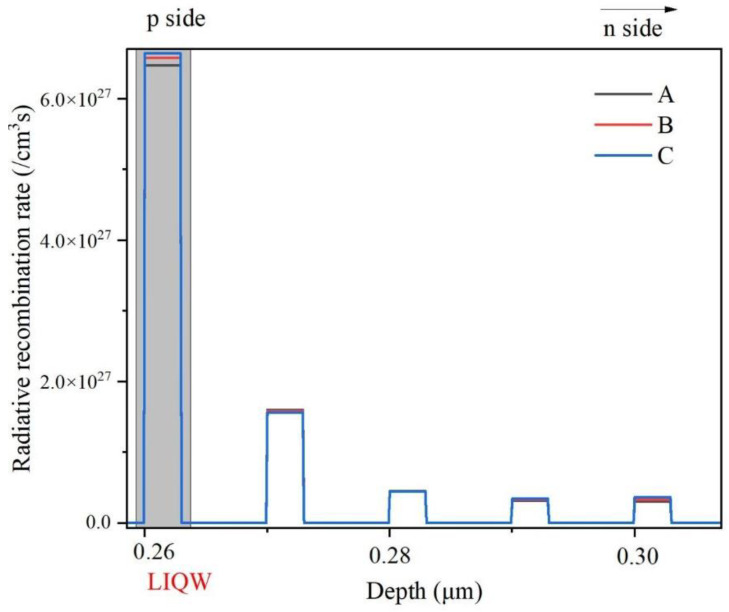
Comparison of radiative recombination rates in the entire MQW active region of all samples under 150 mA injection current. The gray area indicates the position of the LIQW.

To analyze the luminescence properties of different samples in depth, the values of radiative recombination rates of all InGaN QWs at the injection current of 150 mA were extracted and are compared in Figure 3 for all samples. As can be seen, for all samples, the radiative recombination rate in the LIQW is remarkably larger than those in the other InGaN QWs in the MQW active region. This can be ascribed to the fact that most holes may be accumulated in the LIQW, which is near the P-type region, since it is difficult for holes to transfer to these InGaN QWs far from the P-type region due to the large effective mass and low mobility. On the other hand, most electrons with small effective mass and high mobility can readily fly over the entire MQW region and be finally injected into the LIQW (also due to the blocking effect of EBL), causing an accumulation of electrons in the LIQW. As a result, both the holes and electrons are concentrated in the LIQW and thus the radiative recombination process in the LIQW dominate the luminescence emission of yellow MQW samples. 

**Figure 4:**
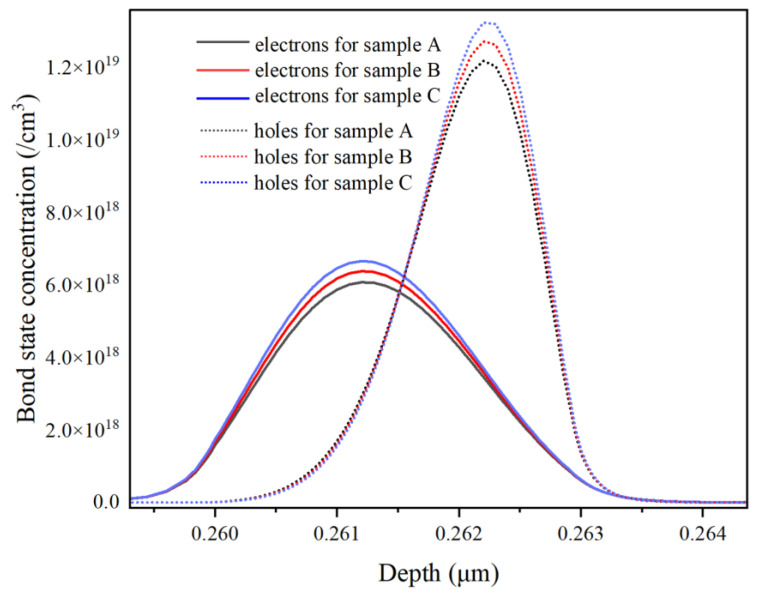
Distributions of electrons (solid line) and holes (dotted line) in the LIQW under 150 mA injection current for all samples.

However, in Figure 3, it is surprising to notice that at 150 mA injection current, the radiative recombination rate in the LIQW is the highest for sample C, while it is the lowest for sample A. This is clearly in contrast to the comparison of IQE and spectral integrated intensity at high injection currents in Figure 2, where sample A’s EL intensity is the strongest. To explore the above contradiction further, the concentrations of electrons and holes in the LIQW are depicted in Figure 4 for all samples at a 150 mA injection current. 

It can be seen that the concentration of electrons and holes in the LIQW monotonically increases from samples A to C. This may be attributed to the increased potential well depth of the LIQW due to the increased In content, since it is difficult for carriers to escape from a deeper quantum potential well formed by the higher-In-content InGaN well layer. In other words, due to the increased In content, the LIQW’s capture ability for both electrons and holes is enhanced, leading to an increased carrier concentration in the LIQW in high-In-content samples. It is well known that if a large number of carriers are injected into InGaN QWs, the influence of polarization electric field in the InGaN well can be partially screened, thus weakening the QCSE and increasing the radiative recombination rates of electrons and holes [22]. Therefore, for sample C, since the concentrations of electrons and holes are the largest in the LIQW, the radiative recombination rate is the highest due to the strongest carrier screening effect in the LIQW, as can be seen in Figure 3. Combining the discussion of Figure 3 and Figure 4, with increasing In content from samples A to C, the radiative recombination rate in the LIQW increases due to the enhanced polarization screening effect induced by the increased carrier concentration in the LIQW due to the enhanced carrier capture ability. In fact, even at low injection currents, the radiative recombination rate is larger for samples with higher In content in the LIQW due to the enhanced carrier capture ability. For example, the peak radiative recombination rates at the injection current of 20 mA are 2.39 × 10^26^, 3.24 × 10^26^, and 4.16 × 10^26^/cm^3^s for samples A, B, and C, respectively. Therefore, in Figure 2, one can see that at low injection currents (below 20 mA), the luminescence efficiency of sample C with the highest In content in the LIQW is the strongest. 

However, it should also be noted that in Figure 2, in contrast to the low-current case, at higher injection currents the luminescence efficiency of samples with higher In content in the LIQW become lower, i.e., the droop effect becomes more serious for the higher In content yellow MQW sample. It is known that under a high injection current (such as 150 mA), the non-radiative Auger recombination and electron leakage may become predominated in the InGaN/GaN MQWs. Thus, the Auger recombination rate in the LIQW and the leakage electron current density which is leaked from the LIQW to the P-type EBL region under a 150 mA current were extracted and compared with the radiative recombination rates in the LIQW for all samples in Table 1.

It can be seen that the radiative recombination rate slightly increases from samples A to C, which has been analyzed in the discussion about Figure 3 and Figure 4, while the Auger recombination rate increases significantly from samples A to C. For instance, compared with sample A, the radiative recombination rate of sample C increases by only 2.2%, but the Auger recombination rate is almost doubled. As is known, the Auger recombination is a main reason for the reduced luminescence efficiency by increasing the non-radiative Auger loss of carriers at high currents, leading to the well-known droop effect [23,24]. As can be seen from Figure 4, the carrier concentrations increase from samples A to C, and hence, the Auger recombination rate increases and accordingly the luminescence efficiency deteriorates at high currents, resulting in an enhanced droop effect. 

Finally, it is also observed that from samples A to C, with increasing In content in the LIQW, the electron leakage current density increases, i.e., the electron leakage becomes more serious. This means that more electrons may fly across the entire MQW active region and directly enter into the P-type GaN region without participating in the radiative recombination process. In fact, it can be seen from Table 1, the difference of leakage currents among three samples are less significant, compared to their Auger recombination rates. Thus, it may not be the main reason responsible for the reduced luminescence efficiency at high currents. However, from samples A to C, the variation trend of leakage currents is consistent with the change of droop behaviors, implying that the increased electron leakage currents are also a factor contributing to the enhanced droop effect. Therefore, the leakage of electrons should be considered and discussed for the analysis on efficiency droop.

**Figure 5:**
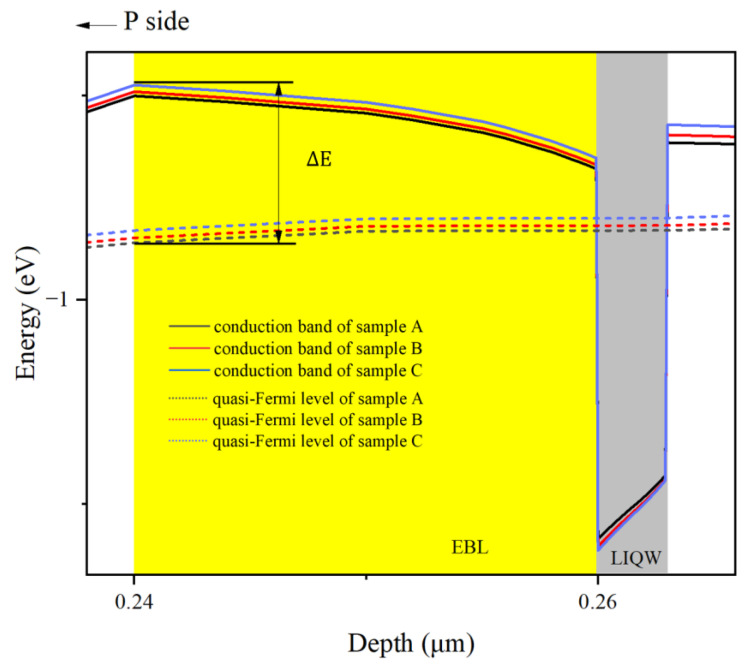
Schematic diagrams of conduction bands (solid line) and electronic quasi-Fermi energy levels (dotted line) near the LIQW under 150 mA injection current for all samples. The positions of the LIQW and AlGaN EBL are indicated by gray and yellow areas, respectively.

To further elucidate this issue, the conduction band diagrams of the LIQW and EBL regions at 150 mA injection current is extracted and compared in Figure 5 for all samples. 

In general, the polarization charge at the interface of InGaN/AlGaN heterojunction not only causes the tilt of the band in the LIQW, but also affects the band in the AlGaN EBL layer. Therefore, as shown in Figure 5, all samples’ conduction bands in the EBL region tilt obviously. It is well known that the tilted energy band in the barrier layer actually increases the potential barrier height and enhances the blocking effect on carrier transport [25,26]. To be convenient, the energy difference ∆E between the highest point of conduction band in EBL region and the electron quasi-Fermi level is defined as the EBL’s effective barrier height for electrons. Theoretically speaking, the increase of In content in the LIQW can lead to an enhanced tilt of the conduction band in the EBL, which in turn gives rise to a higher potential barrier for electrons. Therefore, the barrier height of EBL should be increased from samples A to C. However, the extracted EBL effective barrier heights ∆E of samples A, B, and C are 359, 344, and 328 meV, respectively, i.e., the EBL effective barrier height decreases with increasing In content in the LIQW.

Actually, it can be seen from Figure 5 that the quantum potential well depth of the LIQW becomes deeper from samples A to C, and correspondingly, the carrier capture ability is enhanced. Meanwhile, it should be noted that in Figure 5 from samples A to C, the quasi-Fermi energy level of electrons increases significantly. Therefore, it is reasonable to deduce that because of the enhanced carrier capture ability, there is a larger number of electrons accumulated in the LIQW for the higher-In-content samples, resulting in a significantly lifted electron quasi-Fermi level. As a result, the EBL’s effective barrier height in samples with higher In content in the LIQW is reduced, leading to an increased leakage of electrons.

In brief, the carrier capture ability of the LIQW can be enhanced by increasing In content, and thus, more carriers can be captured in a high-In-content LIQW, leading to an enhanced polarization screening effect, accordingly improved radiative recombination rate, and EL efficiency at low injection currents. However, under the high-injection condition, the carrier concentration in the LIQW increases significantly, causing the significant enhancement of Auger recombination and electron leakage. As a consequence, the luminescence efficiency of samples with higher In content in the LIQW rapidly decreases at high currents, i.e., the droop effect becomes more serious. 

## 4. Conclusions

By increasing the In content in the LIQW layer in yellow-light-emitting InGaN/GaN MQWs, the luminescence efficiency and radiative recombination rate may be improved at low injection currents, which may be ascribed to the enhanced polarization screening effect due to the increased carrier concentration caused by the enhanced carrier capture ability in the LIQW. However, on the contrary, also due to the enhanced carrier capture ability of the LIQW, the Auger recombination and electron leakage become remarkable at high injection currents, which may counteract the beneficial impact of increased radiative recombination rate and finally lead to a significant droop effect for samples with higher In content in the LIQW.

## Figures and Tables

**Table 1:** Comparison of radiative recombination rate, Auger recombination rate, and leakage electron current density in the LIQW under 150 mA injection current for all samples.

Parameters	Sample A	Sample B	Sample C
Radiative recombination rate (/cm^2^s)	2.74 × 10^22^	2.77 × 10^22^	2.80 × 10^22^
Auger recombination rate (/cm^2^s)	4.62 × 10^10^	6.71 × 10^10^	9.34 × 10^10^
Leakage electron current density (A/cm^2^)	3.34	3.58	3.80

## Data Availability

The data presented in this study are available on reasonable request from the corresponding author.
